# The quality of life for students pursuing humanities disciplines and individuals living with HIV during the second wave of the COVID-19 pandemic in Russia

**DOI:** 10.1192/j.eurpsy.2024.1057

**Published:** 2024-08-27

**Authors:** V. V. Titova, V. I. Rozhdestvenskiy, I. A. Gorkovaya, D. O. Ivanov, Y. S. Aleksandrovich

**Affiliations:** Department of Psychosomatics and Psychotherapy, Saint Petersburg State Pediatric Medical University, Saint Petersburg, Russian Federation

## Abstract

**Introduction:**

The COVID-19 pandemic has had a significant impact on people’s lives, affecting various aspects of society and potentially altering the quality of life of certain groups. The World Health Organisation defines quality of life as an individual’s physical, psychological, emotional, and social health as perceived by themselves in relation to society. It appears that the pandemic disproportionately affected the most susceptible societal segments, comprising university students who encountered significant stress due to the shift to remote learning, and individuals living with HIV who faced difficulties in accessing medical assistance.

**Objectives:**

The study aimed to investigate the quality of life of students studying the humanities disciplines and HIV patients during the second wave of the COVID-19 pandemic in Russia.

**Methods:**

Data collection was conducted from January to July 2021, using a Google form developed by the researchers. The study included 35 students from Russian universities studying humanities specialities and 59 HIV-positive patients. To check the quality of life, we used the WHOQOL-BREF questionnaire, adapted for use in Russia.

**Results:**

We found that on the domains “physical and psychological well-being” (M = 20.26±3.89 - students, M = 21.43±3.62 - patients, p = 0.144) and “self-image” (M = 19.11±3.53 vs M = 19.52±2.92, p = 0.553) respondents from the two groups did not differ from each other. The domain “microsocial support” was more pronounced in students than patients (M = 10.71±2.48 vs M = 9.17±2.96, p = 0.011). A similar situation was observed in “social well-being” (M = 27.23±4.33 vs M = 24.97±5.24, p = 0.034).

**Conclusions:**

During the second wave of the COVID-19 pandemic in Russia, individuals living with HIV experienced a lower quality of life compared to students in humanities disciplines. Individuals living with HIV reported lower satisfaction with their relationships within their immediate environment, including family and friends, as well as their overall social well-being, encompassing factors like safety, material wealth, access to medical care, and transportation. In these pandemic conditions, it became evident that individuals with HIV required more extensive social support measures than students.

**Disclosure of Interest:**

None Declared

